# Challenges and opportunities to improve efficiency and quality of prehospital emergency care using an mHealth platform: Qualitative study in Rwanda

**DOI:** 10.1016/j.afjem.2023.07.002

**Published:** 2023-09-20

**Authors:** Mediatrice Niyonsaba, Menelas Nkeshimana, Jean Marie Uwitonze, Justine Davies, Rebecca Maine, Jeanne D'Arc Nyinawankusi, McKenna Hunt, Rob Rickard, Sudha Jayaraman, Melissa H. Watt

**Affiliations:** a Rwanda Biomedical Center, Division of Emergency Medical Services, Rwanda; b University Teaching Hospital of Kigali (Centre Hospitalier Universitaire de Kigali), Rwanda; c University of Birmingham, Institute of Applied Health Research, United Kingdom; d Stellenbosch University, Centre for Global Surgery, Department of Global Health, South Africa; e University of the Witwatersrand, Faculty of Health Sciences, School of Public Health, South Africa; f University of Washington, Department of Surgery, United States; g University of Utah, Honors College, United States; h Rwanda Build Program, Common World Inc., Rwanda; i University of Utah, Department of Surgery, United States; j University of Utah, Department of Population Health Sciences, United States

**Keywords:** Rwanda, Emergency medicine, Pre-hospital emergency care, Qualitative, Ambulance

## Abstract

**Introduction:**

Prompt, high-quality pre-hospital emergency medical services (EMS) can significantly reduce morbidity and mortality. The goal of this study was to identify factors that compromise efficiency and quality of pre-hospital emergency care in Rwanda, and explore the opportunities for a mobile health (mHealth) tool to address these challenges.

**Methods:**

In-depth interviews were conducted with 21 individuals representing four stakeholder groups: EMS dispatch staff, ambulance staff, hospital staff, and policymakers. A semi-structured interview guide explored participants’ perspectives on all aspects of the pre-hospital emergency care continuum, from receiving a call at dispatch to hospital handover. Participants were asked how the current system could be improved, and the potential utility of an mHealth tool to address existing challenges. Interviews were audio-recorded, and transcripts were thematically analyzed using NVivo.

**Results:**

Stakeholders identified factors that compromise the efficiency and quality of care across the prehospital emergency care continuum: triage at dispatch, dispatching the ambulance, locating the emergency, coordinating patient care at scene, preparing the receiving hospital, and patient handover to the hospital. They identified four areas where an mHealth tool could improve care: efficient location of the emergency, streamline communication for decision making, documentation with real-time communication, and routine data for quality improvement. While stakeholders identified advantages of an mHealth tool, they also mentioned challenges that would need to be addressed, namely: limited internet bandwidth, capacity to maintain and update software, and risks of data security breaches that could lead to stolen or lost data.

**Conclusion:**

Despite the success of Rwanda's EMS system, this study highlights factors across the care continuum that could compromise quality and efficiency of prehospital emergency care. Mobile health tools hold great promise to address these challenges, but contextual issues need to be considered to ensure sustainability of use.

**African relevance**

• In low- and middle-income countries (LMICs), emergency conditions account for over half of all years of life lost.

• Africa faces multiple medical emergencies that are aggravated by the weak institutional and health system.

• This paper contributes to the literature by identifying the challenges faced by a relatively new EMS system in Rwanda, and the potential of an mHealth tool to address these challenges.

Alt-text: Unlabelled box

## Introduction

Prompt, high-quality pre-hospital emergency medical services save lives. In the critical moments after trauma or medical emergencies, patients need timely and definitive care to optimize clinical outcomes [1,2]. In high-income countries, highly responsive trauma systems have cut mortality significantly [3]. In low- and middle-income countries (LMICs), emergency conditions account for over half of all years of life lost [4], and the World Bank estimates that 45% of all deaths globally can be prevented with effective emergency care [5], emphasizing the importance of a robust and efficient national emergency medical services (EMS).

A national EMS system typically includes a dispatch center, well-equipped ambulances, and medical personnel, who are together responsible for all pre-hospital emergency care, including triage, medical evaluation, stabilization, and transportation to appropriate care [2,6]. The success of an EMS system depends on both the timeliness of the response, and the quality of the care delivered [5,7,8]. Studies have shown an association between longer prehospital time and patient mortality [8], [9], [10]. However, response time alone is an inadequate predictor of clinical outcomes [10,11]. The focus must simultaneously be on the quality of care in the prehospital setting [12,13], with a focus on a rapid and accurate diagnostic decision-making process in the field, and transportation to the appropriate health facility based on patient needs [14,15].

Prehospital emergency care services face common challenges worldwide, including documentation and communication structures, locating the emergency following a call, provision of appropriate and responsive medical care at the scene, and facilitation of efficient handover from EMS to the most appropriate hospital based on patient needs [16,17]. Electronic or mobile health (mHealth) tools hold promises to address these challenges by supporting the delivery of consistent and high-quality services [18]. In Singapore, the implementation of machine learning tools in dispatch improved the efficiency of the triage process [19]. Digital tools and platforms can help to improve prehospital emergency care by streamlining communication, providing standardized algorithms for clinical decision making, supporting geo-location of emergencies and emergency response teams, and facilitating the collection of data for quality improvement [18,[20], [21], [22]]. This is evidenced by an experimental study in Japan, where a mobile application resulted in prompt and real time data sharing from prehospital to hospital based emergency services [23]. Although mHealh technologies hold great advantages, these can be hampered by challenges related to infrastructure (internet, cost, maintenance, personnel), lack of equipment, and technology constraints [24,25]. Other challenges associated with integrating mHealth technology into routine practice include the acceptability and usability of the technology, the integration of the technology into routine practice, data security and privacy, and reliability of data generated [26].

Rwanda has made progress in health system strengthening, and the current Ministry of Health Strategic Plan (2018–2024) prioritizes building capacity for prehospital and emergency services [27]. Prehospital emergency care throughout Rwanda is led by Service d'Aide Medicale Urgente (SAMU). SAMU operates a national emergency number (“912″) and manages 277 ambulances across the country. While Rwanda has a comprehensive model of pre-hospital emergency care service, the country's latest EMS strategic plan (2018–2024) has highlighted several challenges of the current system, including: limited clinical and non-clinical EMS training, insufficient personnel, absence of a robust monitoring and evaluation system, inability to accurately locate emergency events, inefficient coordination and communication across the EMS system, limited use of technological tools, and limited financial resources [Rwanda EMS Strategic Plan (2018–2024)]. Despite these challenges, the Rwandan EMS is striving to meet its vision “to reduce morbidity and mortality through provision of high quality and integrated pre-hospital emergency care”. This study supports that broader mission by identifying the factors that potentially compromise efficiency and quality of pre-hospital emergency care in Rwanda and exploring opportunities for a mobile health tool to address these challenges.

## Methods

### Overview

This study was conducted in Rwanda by emergency medical service with collaboration of academic partners in the United States (University of Utah and University of Washington) and the United Kingdom (University of Birmingham). The study was part of a larger research project funded by the U.S. National Institutes of Health (R21 TW011636) to develop and pilot test an mHealth tool to improve communication between dispatch, ambulances and receiving hospitals, in order to improve patient outcomes. In this phase of the research, we conducted individual in-depth interviews with stakeholders, in order to understand the strengths and challenges of the system, and the opportunities for an mHealth tool.

### Sample

We recruited 21 individuals for representation across four stakeholder groups: dispatch center (*n* = 5), field ambulance staff (*n* = 5), receiving hospitals (*n* = 8), and policymakers (*n* = 3). Individuals were eligible if they had worked in one of these sectors for at least six months and were actively involved in the management of pre-hospital emergencies. Many participants worked across multiple sectors (e.g., someone might work shifts in both dispatch and ambulance). We identified eligible participants based on workplace rosters and consulted with SAMU leadership to purposively recruit individuals who would provide good insight about the strengths, challenges and opportunities of the current EMS structure.

### Procedures

The SAMU leadership introduced the study to the staff. The research staff then reached out directly to individuals via email or phone to invite them to participate in an interview. Potential participants were informed that they could choose to participate or not, without any repercussions on their employment, that they could withdraw their participation at any time, and that identified data would not be shared with their employers. All identifying information was stored separately from the participants’ data, and all transcripts were anonymized prior to storing on our research servers.

Following informed consent, interviews were conducted at a time and place convenient for the participant. Given restrictions of the COVID-19 pandemic, some interviews (5 of the 21) were conducted via Zoom. Interviews were conducted by the first author (MN), who has experience working in pre-hospital emergency care in Rwanda. She was trained and supervised by MHW, a researcher with qualitative expertise. Interviews were conducted in the language that participants felt most comfortable conversing in (English, French or Kinyarwanda) and lasted on average one hour (range: 28 min to over 4 h). Interviews were audio-recorded with participant consent, and subsequently transcribed and translated into English by project staff.

The semi-structured interview guide had three primary sections: perspectives on the continuum of pre-hospital emergency care, ideas for how the current system could be modified to improve the quality and efficiency of care, and feedback on the potential utility of mHealth tools to address existing challenges. The guide was structured with broad open-ended questions, followed by specific probes to elicit detailed information and examples. The guide was tailored to the stakeholder group and covered the areas specific to each group in more detail. Data analysis was iterative to ensure that our target sample provided data saturation on our key research questions.

### Data analysis

A structured codebook was developed to facilitate coding of descriptive information and emerging themes that arose from the interviews. Codes were defined for each of the points along the continuum of pre-hospital emergency care (e.g., triaging calls at dispatch, locating an emergency, handover to hospital). Emerging themes were identified under several domains, including system challenges, mHealth tool advantages, mHealth tool capabilities, mHealth tool challenges, and mHealth tool implementation. The codebook was developed following a thorough reading of the transcripts and discussion with the investigator team, and was refined during the coding process to reflect new insights into the data. A single analyst (MHW) coded the transcripts using NVivo Version 12, in consultation with the first author (MN). The coded data were retrieved and synthesized in an Excel document, with the goal of describing factors along the care continuum that affect the efficiency and quality of pre-hospital emergency care and how an mHealth tool might address those factors. The findings were discussed among the multi-national and inter-disciplinary team (including clinicians and researchers from Rwanda, the United States, and the United Kingdom) in order to ensure that the data were accurately interpreted and contextualized.

### Trustworthiness of data

To maximize the trustworthiness of the qualitative data, the data collection process was facilitated by a semi-structured guide, and interviews were audio recorded and subsequently transcribed. Analysis was conducted by an established qualitative researcher, and other members of the multi-disciplinary team contributed to consensus building on findings. SAMU leadership reviewed the data as a “member checking” process to ensure that findings were properly interpreted, contextualized, and generalizable to the EMS in Rwandan context.

### Ethical statement

The study was approved by the University Teaching Hospital of Kigali Ethics Committee (Protocol EC/CHUK/1/077/2021). All participants provided written informed consent prior to participation.

## Results

All individuals who were invited agreed to participate. We interviewed 21 participants who represented policymakers (*n* = 3), hospital staff (*n* = 8), and SAMU staff (*n* = 10) who work across dispatch and ambulances. The participants had on average 6.9 years (Range: 9 months to 20 years) of experience working with EMS.

### Factors that compromise the efficiency and quality of pre-hospital emergency care

**Fig. 1:**
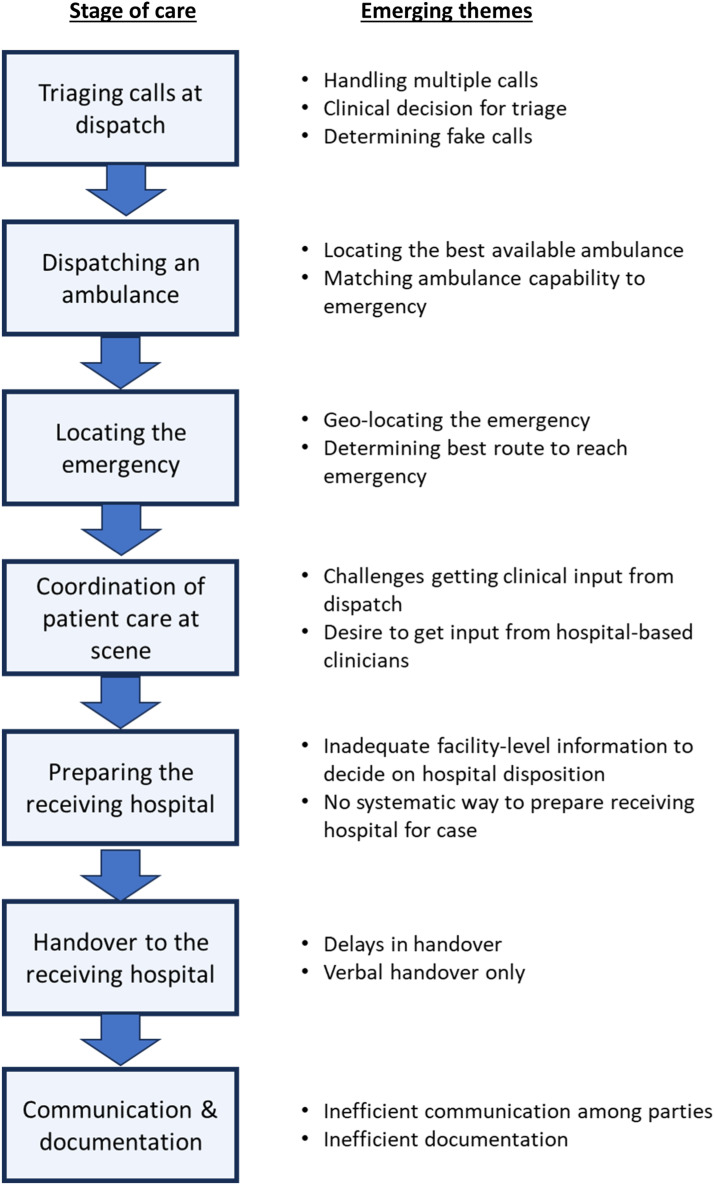
Summary of themes related to factors that compromise the efficiency and quality of care across the prehospital emergency care continuum.

The themes that emerged in the qualitative data reflect factors that compromise the efficiency and quality of pre-hospital emergency care across seven stages of the continuum of care: 1) triaging calls, 2) dispatching an ambulance, 3) locating an emergency, 4) coordination of patient care at scene, 5) preparing the receiving hospital, 6) handover to the receiving hospital, and 7) communication and documentation. Fig. 1 summarizes the 15 themes that emerged across these seven stages. Below we summarize these emerging themes and share representative quotes.

### Stage 1: triaging calls at dispatch

Within this stage, we identified three themes. First, dispatch staff were often faced with handling multiple calls at once and were hampered by resource constraints of only two staff members and two cell phones to field calls.

“We may have 5 calls at the same time but we only have two cell phones so you can miss some information because you can't attend two calls at the same time.” (Dispatch and ambulance staff)

Second, dispatch staff lacked clear clinical decision algorithms across various emergency situations.

“When we receive a call we try to collect different information especially on patient major complaints, we analyze degree of severity, the problem the patient may have, from that is where we say this might be moderate or severe emergency and that there might be a real need of ambulance or not.” (Dispatch and ambulance staff)

Third, staff were encumbered with the burden of determining hoax calls, which often outnumbered valid calls.

“For 24 h, on average it's around 50 valid calls. But we can't forget that we have hundreds and hundreds of fake calls that are even disturbing dispatching team to receive the real/valid calls.” (Dispatch staff)

### Stage 2: dispatching the ambulance

Within this stage, we identified two themes. First, dispatch was hampered in locating the available ambulance, because they lacked geo-tracking of ambulance location.

“If you want to talk about tracking the ambulances, we don't have that… When you work at call center, the most important skills you should develop, it is knowing where your teams are by keeping talking to them.” (Dispatch and ambulance staff)

Second, dispatch struggled with matching the emergency to the ambulance capability, including the vehicle characteristics (e.g., off road capability), the equipment on-board, and the current staffing of the ambulance.

“Our ambulances are not able to reach all locations. Some can't be driven in bad roads, cannot tolerate slippery area. So, in that case, we will think also on capability of ambulance to send.” (Dispatch and ambulance staff)

### Stage 3: locating the patient with the emergency

At this stage, we identified two themes. First, the teams struggled with geo-locating the patient, given the reliance on verbal directions and local knowledge.

“The most critical issue is geolocation of patients. Sometimes teams get lost on the way and sometimes the callers don't know the place to guide the dispatch team. And the latter does not have technological capacity to geolocate.” (Policymaker)

Second, the teams did not have a systematic way to determine the best route to the location, based on dynamic traffic patterns and road conditions.

“It's very difficult to find the (best) road that brings you to emergency scene, and in that situation it is difficult to orient the team.” (Dispatch staff)

### Stage 4: coordinating patient care at scene

At this stage, we identified two themes. First, ambulance staff have protocols to help stabilizing and assessing patients, but often needed additional clinical input from dispatch to appropriately manage the case. In some cases, communication delays led to delays in providing emergency care for patients.

“We may arrive to the scene and find out that our ideas are not enough to save the patient then we call the dispatch center for advice.” (Ambulance staff)

Second, ambulance staff desired input from hospital-based clinicians on managing cases, but there was no direct mechanism to support that communication.

“It would be good if we could share patient information directly with the ED doctor, he could even advise on additional procedures/treatment while on field and on the way.” (Dispatch and ambulance staff)

### Stage 5: preparing the receiving hospital

At this stage, we identified two themes. First, dispatch noted that they had inadequate facility-level information to decide on hospital disposition, including available beds, specialists, and diagnostic equipment.

“We do not have tools to know whether they have beds or not, but we communicate by calling them.” (Dispatch and ambulance staff)

Second, there was no systematic way to prepare the receiving hospital for the case aside from relying on dispatch to call the hospital and verbally recount the case.

“When our reception phone is off and on charger, they will call us, but we are not able to pick the call. Or when the physician is not responding immediately because he/she is busy, and the resident who receive the call is a junior resident. The decision making takes time or can take a wrong decision like telling to bring the patient in another hospital while the patient really needs to be here and at the end the patient will come from that hospital to [here] later.” (Hospital staff)

### Stage 6: patient handover to the hospital

At this stage, two themes emerged. First, the ambulance staff often faced delays in handing over the patient to hospital staff given challenges of over-crowding and resource constraints.

“[Delays] will depend if I can say, on the capability or capacity for the facilities to take patients. We may find sometimes that there is an issue of limited places or the hospital staff are attending another critical case.” (Dispatch staff)

Second, handover was done solely by verbal communication, which participants noted may contribute to inaccurate or incomplete information about the patient's care in the pre-hospital period.

“We wish to have a written handover because you can forget the information given, but if you have the written handover, it will help.” (Hospital staff)

### Stage 7: communication and documentation

Across the entire pre-hospital care continuum, we identified two cross-cutting themes related to communication and documentation. First, there was a pervasiveness of inefficient communication among parties, namely among the caller, dispatch, ambulance, and hospital.

“There is a gap of the inter-operability of EMS, hospitals, and the caller, especially on the flow of information. this is playing a critical role in the time of response in a negative way.” (Policymaker)

Second, there were inefficiencies in the documentation of the clinical experience. These inefficiencies in communication and documentation contributed to slowing down the care process and potentially also introduced gaps in the quality of care.

“We have a patient file where we record. Sometimes I record information on the phone then I put it later on the file. While we are at the scene, I may also ask the call center to keep some information like vital signs because I need that while handing over the patient to the receiving hospital.” (Ambulance staff)

### Opportunities for a mobile health tool to address challenges

Stakeholders identified four areas where an mHealth tool could improve the efficiency and quality of pre-hospital emergency care: 1) efficient location of the emergency, 2) streamlining communication for decision making, 3) documentation with real-time communication, and 4) routine data for quality improvement. They also acknowledged several challenges of implementing mHealth technologies.

### Efficient location of the emergency

Challenges locating the scene of the emergency were seen as a leading cause of delay, and an mHealth tool was identified as a possible solution.

“There is a considerable time lost while trying to identify where the patient is. If the dispatching team can get that option to geo locate the patient and this platform is easily accessible by field teams, this can easily change the way the operations are running now.” (Dispatch and policy staff)

### Streamline communication for decision making

Participants noted the challenge of multiple calls in order to share information and make decisions. They pointed to the potential for an mHealth tool to streamline communication for decision making by linking multiple stakeholders.

“The mobile team can have tablets installed in the ambulance so that information received at the call center can be easily, quickly, effectively and automatically shared with the team we want to dispatch. An mHealth tool can help make our intervention not delay because it will easily connect with the 4 stakeholders: the caller, the command center, field team and receiving health facility.” (Communication and dispatch staff)

### Documentation with real-time communication

Given the inefficiencies in documentation and communication, an mHealth tool was noted as one possible way to share data in real time. This could include ambulance data (e.g., location, capabilities), hospital data (e.g., available beds, specialty services), and patient data (e.g., patient history, vital signs, clinical care).

“At the dispatch center, it is possible to follow the movement of the ambulance and track the ambulance departure, arrival at the scene of the incident, but also the arrival at the receiving hospitals. In addition to these, to have effective communication with the receiving facilities, be aware of the availability of the space at the hospital, availability of specialized services like CT scan, specialist or specialized care. I think we can communicate with the receiving facilities at the same time with people at the scene of emergency.” (Policymaker)

### Routine data for quality improvement

EMS data are currently being recorded on paper and subsequently entered into databases. Stakeholders felt that if data were collected consistently and in real-time, then regular reports could be generated to inform quality improvement initiatives.

“I think for quality improvement purpose, having an electronic platform could easily allow SAMU leadership team to have regular checks on data and can allow them to take critical decisions to influence the policy change in order to adapt to the current trends. So, we can't have policy change if we don't have data.” (Policymaker)

### Challenges of implementing mHealth technologies

While stakeholders identified advantages of an mHealth tool, they also mentioned challenges that would need to be adequately addressed during the development and implementation phases. The following concerns emerged: limited internet bandwidth to consistently use the technology, capacity to maintain and update any software, and risks of data security breaches that could lead to stolen or lost data.

“I am not an expert in IT, but I would anticipate that any IT technical issues would hamper the way we operate and oblige us to return the old system. I also mentioned about cyber-security, if we get hacked and the sensitive information about patients are robbed by hackers… Another challenge is to have experts to maintain the system. Because, I think having a system running is good, but to maintain this application or the platform will be somehow critical.” (Policymaker)

## Discussion

This study sought feedback from a range of stakeholders across the EMS system in Rwanda on factors that compromise the quality and efficiency of pre-hospital emergency care, and the potential for mHealth tools to address these. Despite the success of the Rwandan health sector in general and the EMS in particular, the data in this study highlighted factors across the care continuum that limit the impact of the EMS system and identified opportunities of an mHealth tool to address existing challenges.

Across all phases of the pre-hospital emergency care continuum, inefficiencies in documentation and communication were mentioned by participants. Documentation is typically done on paper and later entered into a computer database. It is not shared in real-time with other stakeholders to support decision-making. Inefficient communication can compromise the quality of care given to a patient and delay quality improvement [28,29]. Participants discussed how communication across the four stakeholder groups (caller, dispatch, ambulance, hospital) is often disjointed and relies on verbal handover of information, which has the potential to miss key information, especially in the midst of hectic emergency scenarios or multiple calls at once. The existing communication system puts dispatch at the epicenter of all communication channels, meaning that other parties (e.g., the caller and the ambulance team, or the ambulance team and the hospital) do not directly communicate with each other. The same challenges have been reported by other studies, where communication difficulties lead to conflicts between dispatch and other operations with increase of legal risks [30], [31], [32]. Participants believed that an mHealth tool could potentially solve this problem by automatically connecting the four entities at the same time and making the information sharing and documentation easier, faster, and more efficient. They also perceived the potential for an mHealth tool to systematically collect and record data that could later be used for quality improvement initiatives, as demonstrated in other studies [33,34].

Participants discussed a pervasive challenge of locating the person in need of emergency medical support. Delays in locating the patient can increase the risk of severe morbidity and mortality[10,35]. Our team has explored this challenge of locating the site of emergency in Rwanda in a separate publication [36]. An mHealth tool that collects the geolocation of the emergency from the caller can support the timely arrival of the ambulance to the scene [37], as has been demonstrated in other settings [38]. A tool that combines the location of the emergency with the location of ambulance fleet and potential referral hospitals would greatly improve the timeliness of care [39,40].

Stakeholders raised an issue of triaging calls at dispatch, where they are stressed by a large number of callers, including both real and prank calls, as well as a lack of algorithms to guide triage. This high burden in triage may lead to inappropriate decisions, such as misclassifying emergency cases and causing misuse of resources. The same problem has been reported in other EMS settings [31,32]. Stakeholders also perceived the importance of decision-making algorithms and guidelines for triage and field management at the site of emergencies, in order to maximize the quality of care. Algorithms are important across multiple phases of the pre-hospital continuum, including dispatch, on-site care, and referral to hospital [41]. The presence of decision-making algorithms, together with clear documentation and communication systems among stakeholders, can assure that clinical decisions are aligned with evidence-based practices. While decision support tools are helpful in all clinical settings, they may be particularly valuable in the pre-hospital environment, given the focus on prompt stabilization and referral [42].

The efficient deployment of appropriate ambulance services is key to ensuring prompt and proper patient care. Participants pointed out that the dispatching of ambulances is challenging given the lack of tracking devices on vehicles and an inability to determine the best route to an emergency, leading to delays of response and affecting not only the patients but also the physical and mental health of dispatch personnel [43]. Participants shared that an mHealth tool, informed by user centered design, can support dispatch in deploying the proper ambulance and shorten the transport time to the scene of the emergency [44,45].

The impact of an EMS system is dependent on proper handover to a health facility, where the patient can receive timely and appropriate care [46]. The perspectives of stakeholders suggest specific gaps in the bridge between EMS and the hospital. From the perspective of the dispatch, there is no consistent information about the hospital's capacity to receive and treat a patient. From the perspective of the hospital, they do not receive complete information about a patient's condition, which limits their ability to prepare for the patient's arrival. The challenges in coordination and communication can hamper the quality of services [32,46], but can be improved by implementing a standardized procedure for patient handover [47]. Improved communication with hospitals can not only improve efficient decision making, but can also advance the integration of the hospital system into the EMS infrastructure in order to improve patient outcomes [29].

This study highlights the potential of mHealth tools to address challenges within the EMS care continuum in LMIC health systems. Adoption of mHealth technologies has been low in the African Region; a WHO survey found that only 40% of countries used any mHealth resource to manage emergency situations [48]. The survey report attributes these low uptake rates to insufficient infrastructure in these countries such as “lack of paved roads, [and] dispatch systems connecting ambulances to hospitals” [48]. In high income country settings, there is strong evidence that mHealth technology can serve as a platform for communication and care coordination [45]. For adoption of these technologies in LMICs such as Rwanda, careful contextualization during design and testing is paramount to avoid unanticipated negative externalities [49].

### Study limitation

Our study sought perspectives of multiple stakeholder groups involved in the EMS system in Rwanda, but had some limitations that must be considered. First, the interviews were subject to social desirability bias; the interviewers were part of the broader EMS infrastructure and participants may have been cautious to speak freely about their opinions on the system. Second, this study does not include the perspective of other important stakeholders, most notably community users of the national EMS system and other emergency response actors (e.g., police). Our study was conducted solely in Kigali, and may have missed the unique challenges of providing prehospital emergency care in rural areas of the country. Finally, this study did not seek input from IT personnel to understand the current digital infrastructure and the feasibility and barriers of introducing an mHealth technology. Future studies should seek out these perspectives.

## Conclusion

A robust EMS system can prevent unnecessary morbidity and mortality, while preserving scarce health care resources. In this paper, stakeholders of the Rwanda EMS system highlighted several addressable challenges to improve the quality and efficiency of prehospital emergency care. Most notable are efficiencies in communication and documentation, geolocation of emergencies, and handover from EMS to hospital. An mHealth tool might be able to address some of these challenges to improve the efficiency and quality of EMS care in Rwanda and in turn improve clinical outcomes. Addressing specific challenges such as IT support and infrastructure, cybersecurity and internet bandwidth are essential to successful implementation and sustainability of any context appropriate tool. Rwanda has one of the most advanced EMS systems on the continent, and therefore offers a unique opportunity to develop and test these tools so that they can be scaled up to other African EMS systems.

## Dissemination of the results

The findings of this study was presented to the staff of Rwanda emergency medical service. A report of findings was given to policy makers (Division manager of SAMU and Prehospital Team Leader).

## Authors Contribution

Authors contributed as follow to the conception, research, drafting and revising the work critically for important intellectual content: MN contributed 35%; MHW and SJ contributed 15% each; MN, JMU, JD, RM, JDN, MH and RR contributed 5% each. All authors approved the version to be published and agreed to be accountable for all aspects of the work.

## Declaration of Competing Interest

This study was funded by a grant from the U.S. National Institutes of Health (R21 TW011636, PI Jayaraman). Justine Davies was supported by a grant from the United Kingdom Research and Innovation for Global Health Transformation (RIGHT) Programme (NIHR203062, PI Davies). The following authors were employed by the Rwandan Service d'Aide Medicale Urgente (SAMU) at the time of the research: Mediatrice Niyonsaba, Jean Marie Uwitonze, and Jeanne D'Arc Nyinawankusi. We have no other conflicts of interest to report.

## Appendix Supplementary materials

Image, application 1
