# Supplementary material for: Challenges and opportunities to improve efficiency and quality of prehospital emergency care using an mHealth platform: Qualitative study in Rwanda
Source: Afr J Emerg Med. 2023 Sep 20;13(4):250–7. doi: 10.1016/j.afjem.2023.07.002 (PMC10520315; doi:10.1016/j.afjem.2023.07.002)
Supplement: Supplementary file 1 [file mmc1.pdf]

# Défis et opportunités pour améliorer l'efficacité et la qualité des soins pré-hospitaliers d'urgence à l'aide d'une plateforme « mHealth » : étude qualitative au Rwanda


**RESUME**

**Introduction :** Les services médicaux d'urgence pré-hospitaliers rapides et de haute qualité peuvent réduire considérablement la morbidité et la mortalité. Le but de cette étude était d'identifier les facteurs qui compromettent l'efficacité et la qualité des soins d'urgence pré-hospitaliers au Rwanda, et explorer les opportunités de relever ces défis par un outil de santé mobile, le « mHealth ».

**Méthodes:** Des entretiens approfondis ont été menés avec 21 personnes représentant quatre groupes qui prirent part à cette étude: personnel du centre de régulation médicale urgente, les ambulanciers, le personnel hospitalier et les décideurs politiques. Un guide d'entrevue semi-structuré a exploré les points de vue des participants sur tous les aspects de la continuité des soins pré-hospitaliers, de la réception d'un appel par le centre de régulation jusqu'à la prise en charge à l'hôpital. On a demandé aux participants comment le système actuel pourrait être amélioré et l'utilité potentielle d'un outil « mHealth » pour relever les défis existants. Les entrevues ont été enregistrées sur bande audio et les transcriptions ont été analysées thématiquement avec NVivo.

**Résultats:** Les intervenants ont identifié les facteurs qui compromettent l'efficacité et la qualité des soins dans la continuité des soins pré-hospitaliers d'urgence : triage à la régulation, l'envoi des ambulances appropriées, localiser l'urgence, coordonner les soins des patients sur place, informer les hôpitaux d'accueil et la remise du patient. Ils ont identifié quatre domaines dans lesquels le « mHealth » pourrait améliorer les soins : localisation efficace de l'urgence, organiser la communication pour la prise de décisions, documentation avec communication en temps réel et données de routine pour l'amélioration de la qualité. Bien que les parties prenantes aient identifié les avantages d'un outil « mHealth », elles ont également mentionné les défis qui devraient être résolus, à savoir : bande passante d'internet limitée, capacité d'entretien du logiciel et sa mise à jour, et les risques de failles dans la sécurité des données pouvant entraîner leur vol ou leur perte.

**Conclusion:** Malgré le succès des Services d'Aide Médicale Urgente du Rwanda, cette étude met en évidence des facteurs qui, dans la continuité des soins pré-hospitaliers d'urgence, pourraient compromettre leur qualité et leur efficacité. Les outils de santé mobiles sont très prometteurs pour relever ces défis, mais les problèmes contextuels doivent être pris en compte pour assurer la pérennité de leur utilisation.
